# The relative and attributable risks of cardia and non-cardia gastric cancer associated with *Helicobacter pylori* infection in China: a case-cohort study

**DOI:** 10.1016/S2468-2667(21)00164-X

**Published:** 2021-11-25

**Authors:** Ling Yang, Christiana Kartsonaki, Pang Yao, Catherine de Martel, Martyn Plummer, Daniel Chapman, Yu Guo, Sarah Clark, Robin G Walters, Yiping Chen, Pei Pei, Jun Lv, Canqing Yu, Rima Jeske, Tim Waterboer, Gary M Clifford, Silvia Franceschi, Richard Peto, Michael Hill, Liming Li, Iona Y Millwood, Zhengming Chen

**Affiliations:** a Clinical Trial Service Unit & Epidemiological Studies Unit, Nuffield Department of Population Health, University of Oxford, Oxford, UK; b Medical Research Council Population Health Research Unit, Nuffield Department of Population Health, University of Oxford, Oxford, UK; c Early Detection, Prevention, and Infections Branch, International Agency for Research on Cancer, Lyon, France; d Department of Statistics, University of Warwick, Coventry, UK; e Chinese Academy of Medical Sciences, Dong Cheng District, Beijing, China; f Department of Epidemiology and Biostatistics, School of Public Health, Peking University Health Science Centre, Beijing, China; g Infections and Cancer Epidemiology Division, German Cancer Research Centre, Heidelberg, Germany; h Centro di Riferimento Oncologico IRCCS, Aviano, Italy

## Abstract

**Background:**

*Helicobacter pylori* infection is a major cause of non-cardia gastric cancer (NCGC), but its causal role in cardia gastric cancer (CGC) is unclear. Moreover, the reported magnitude of association with NCGC varies considerably, leading to uncertainty about population-based *H pylori* screening and eradication strategies in high-risk settings, particularly in China, where approximately half of all global gastric cancer cases occur. Our aim was to assess the associations of *H pylori* infection, both overall and for individual infection biomarkers, with the risks of NCGC and CGC in Chinese adults.

**Methods:**

A case-cohort study was done in adults from the prospective China Kadoorie Biobank study, aged 30–79 years from ten areas in China (Qingdao, Haikou, Harbin, Suzhou, Liuzhou, Henan, Sichuan, Hunan, Gansu, and Zhejiang), and included 500 incident NCGC cases, 437 incident CGC cases, and 500 subcohort participants who were cancer-free and alive within the first two years since enrolment in 2004–08. *H pylori* biomarkers were measured in stored baseline plasma samples using a sensitive immunoblot assay (HelicoBlot 2.1), with adapted criteria to define *H pylori* seropositivity. Cox regression was used to estimate adjusted hazard ratios (HRs) for NCGC and CGC associated with *H pylori* infection. These values were used to estimate the number of gastric cancer cases attributable to *H pylori* infection in China.

**Findings:**

Of the 512 715 adults enrolled in the China Kadoorie Biobank between June, 2004, and July, 2008, 500 incident NCGC cases, 437 incident CGC cases, and 500 subcohort participants were selected for analysis. The seroprevalence of *H pylori* was 94·4% (95% CI 92·4–96·4) in NGCG, 92·2% (89·7–94·7) in CGC, and 75·6% (71·8–79·4) in subcohort participants. *H pylori* infection was associated with adjusted HRs of 5·94 (95% CI 3·25–10·86) for NCGC and 3·06 (1·54–6·10) for CGC. Among the seven individual infection biomarkers, cytotoxin-associated antigen had the highest HRs for both NCGC (HR 4·41, 95% CI 2·60–7·50) and CGC (2·94, 1·53–5·68). In this population, 78·5% of NCGC and 62·1% of CGC cases could be attributable to *H pylori* infection. *H pylori* infection accounted for an estimated 339 955 cases of gastric cancer in China in 2018.

**Interpretation:**

Among Chinese adults, *H pylori* infection is common and is the cause of large numbers of gastric cancer cases. Population-based mass screening and the eradication of *H pylori* should be considered to reduce the burden of gastric cancer in high-risk settings.

**Funding:**

Cancer Research UK, Wellcome Trust, UK Medical Research Council, British Heart Foundation, Kadoorie Charitable Foundation, National Key Research and Development Program of China, and National Natural Science Foundation of China.

## Introduction

Gastric cancer is the fifth most frequently diagnosed cancer and the second leading cause of cancer death globally, causing more than 1 million new cases and approximately 770 000 deaths in 2020, with China alone accounting for approximately half (478 000) of the number of global new cases.^1^ One of the most notable and preventable causes of gastric cancer is *Helicobacter pylori* infection, which caused an estimated 0·8 million new gastric cancer cases globally in 2018, based mainly on relative risk (RR) estimates from studies in populations from Europe, the USA, and Australia.^2^

The prevalence of *H pylori* infection varies greatly between and within countries, from 20% to 50% in high-income countries to more than 80% in many low-income countries.^3^ Although several epidemiological studies have consistently shown the strong association of non-cardia gastric cancer (NCGC) with *H pylori* infection in diverse populations, the reported RR estimates varied considerably between the studies.^4^, ^5^, ^6^ Moreover, there is substantial uncertainty about the role of *H pylori* infection in the cause of cardia gastric cancer (CGC), which accounts for approximately a third of gastric cancer cases globally. By contrast to a few east Asian studies showing a positive association of CGC with *H pylori* infection,^7^, ^8^ albeit more modest than that for NCGC, most studies of populations in Europe, the USA, and Australia have reported either null or reduced risks of CGC associated with *H pylori* infection.^5^, ^9^

**Research in context**

**Evidence before this study**

We searched for articles in PubMed published in English, Chinese, and other languages (including Japanese and Korean with English abstracts available, but with a focus on papers published in English) from Jan 1, 1990, to Dec 8, 2020, for individual studies or pooled or meta-analyses on *Helicobacter pylori* infection and risks of gastric cancer. The search terms (“*H. pylori*” or “*Helicobacter pylori*”) and (“gastric” or “non-cardia” or “cardia” or “stomach”) and (“cancer” or “carcinoma”) and (“risk” or “association”) were used. Overall, we identified six pooled analyses or meta-analyses that included approximately 20 prospective studies and a larger number of retrospective studies, with approximately a third involving east Asian populations. For non-cardia gastric cancer (NCGC), most studies have consistently shown a positive association with *H pylori* infection, but the relative risk (RR) estimates varied by more than 20 times, partly because of differences in study design, assay methods used, and exclusion criteria applied to reduce the effects of gastric atrophy on *H pylori* seropositivity. In a pooled analysis undertaken by the Helicobacter and Cancer Collaborative Group that included 12 prospective studies, *H pylori* infection as established by ELISA was associated with a 3·0-fold (95% CI 2·3–3·8; n=762 cases) increased risk of NCGC, with a higher RR (5·9, 95% CI 3·4–10·3; n=223 cases) after restricting analyses to cases with blood samples collected over 10 years before cancer diagnosis. More recently, in a pooled analysis of three nested case-control studies of populations in Europe, the USA, and Australia using a more sensitive immunoblot assay (HelicoBlot 2·1) for *H pylori* infection, the reported RR was 17·0 (11·6–25·0; n=189 cases) for NCGC, much higher than that obtained using ELISA in the same study population. Previous studies of *H pylori* infection and cardia gastric cancer (CGC), which accounts for approximately 30% of gastric cancer, have produced conflicting findings, especially between studies in east Asia and Europe, the USA, and Australia. In the aforementioned pooled analysis by HCCG (n=274 CGC cases) and a subsequent meta-analysis of 34 studies that also included many retrospective studies, *H pylori* infection showed either a null or negative association with CGC in populations in Europe, the USA, and Australia, but a positive, albeit more modest compared with NCGC, association in east Asian populations. In mainland China, where approximately half of all global gastric cancer cases currently occur, only four prospective studies have examined the associations of *H pylori* infection with gastric cancer. None have used immunoblot assays, excluded cases diagnosed within the first few years after sample collection, or covered multiple diverse areas. Although they showed positive associations of *H pylori* infection with overall gastric cancer, or with NCGC and CGC if available, there was substantial uncertainty about the magnitude of the associations.

**Added value of this study**

Our study had a case-cohort design within the large prospective China Kadoorie Biobank of 0·5 million adults recruited from ten geographically diverse areas in China. In addition to the inclusion of many adjudicated NCGC and CGC cases that occurred at least 2 years after sample collection, it also used a sensitive immunoblot assay to detect *H pylori* infection. Our study showed that *H pylori* infection was associated with 6-times higher risk of NCGC and 3-times higher risk of CGC.These RR estimates were approximately twice those previously reported among Chinese adults. We further estimated that approximately 80% of cases of NCGC and more than 60% of cases of CGC currently occurring in China each year could be attributable to *H pylori* infection. In a combined analysis of the present study with published data from three studies in Europe, the USA, and Australia that used the same HelicoBlot assay, the pooled RR for NCGC was 8·95 (95% CI 5·59–14·33), which can inform the future estimation of the disease burden associated with *H pylori* infection regionally and globally.

**Implications of all the available evidence**

The reliable estimation of the risk and burden of gastric cancer due to *H pylori* infection will help policy makers to develop and implement suitable strategies for cancer prevention locally and globally. The new evidence from this study should help to refine the estimation of the global gastric cancer burden attributable to *H pylori* infection. More importantly, our findings, combined with evidence from a randomised controlled trial of the effects of *H pylori* treatment on gastric cancer risks in China, suggest that population-based *H pylori* screening and eradication should be considered as a key strategy for gastric cancer prevention, before considering mass gastric cancer screening by barium photofluorography or endoscopy in China and other high-risk settings.

As a result of chronic *H pylori* infection, a high proportion of people with gastric cancer might develop severe gastric atrophy several years before cancer development,^10^ which could reduce antibody concentrations and substantially underestimate the risk of *H pylori* infection in case-control studies or prospective studies with a short follow-up, in which *H pylori* infection is measured after or shortly before cancer diagnosis.^5^ Moreover, the risk estimates might also be affected by the sensitivity of the assay used to measure *H pylori* antibodies. In populations in Europe, the USA, and Australia, the immunoblot assay has proven to be more sensitive than conventional ELISA to detect *H pylori* antibodies, with some studies reporting 2–5 times higher risks for NCGC with an immunoblot than with ELISA in the same study populations.^6^, ^11^, ^12^, ^13^ However, these previous studies tended to be small, typically involving fewer than 100 patients with cancer. To our knowledge, no large prospective study using an immunoblot assay has been done in China where *H pylori* infection is highly prevalent, with different *H pylori* strain-specific features from populations in Europe, the USA, and Australia.^14^

Using an immunoblot assay and a case-cohort study design within the prospective China Kadoorie Biobank study of more than 0·5 million adults from ten geo-graphically diverse areas, we aimed to assess the associations of *H pylori* infection, both overall and for individual infection biomarkers, with risks of NCGC and CGC in Chinese adults. We also estimated the number of gastric cancer cases attributable to *H pylori* infection in China.

## Methods

### Study population

Details of the design, methods, and study participants in the China Kadoorie Biobank study have been previously described.^15^ Briefly, the baseline survey was done between June, 2004, and July, 2008, in ten (five urban and five rural) geographically diverse areas across China (Qingdao, Haikou, Harbin, Suzhou, Liuzhou, Henan, Sichuan, Hunan, Gansu, and Zhejiang) and enrolled 512 715 adults aged 30–79 years. The ten regions were selected with the aim to maximise the diversity from the geographical locations, socioeconomic characteristics, risk exposures, and disease patterns, while considering the quality of death and disease registries and local capacity. In each study area, a regional coordinating centre and survey team were set up and all permanent residents aged 30–79 years identified from local residential records were invited to participate. Extensive data were collected through a laptop-based questionnaire (eg, sociodemographic, lifestyle and dietary factors, and medical history) and physical measurements (eg, blood pressure and adiposity), along with the collection of a 10 mL blood sample for long-term storage. Ethics approval was obtained from relevant international, national, and local ethics committees (in the UK, Oxford Tropical Research Ethics Committee; in China, the Chinese Centre for Disease Control and Prevention Ethical Review Committee and the Chinese Academy of Medical Sciences and Peking Union Medical College Ethical Committee). All participants provided written informed consent.

Follow-up of participants in the China Kadoorie Biobank study was done through linkage, via unique personal identification numbers, to established mortality and morbidity registries (for cancer, stroke, ischaemic heart disease, and diabetes) that were already available in the study areas since the beginning of the baseline survey, and to the nationwide health insurance system, which records any episodes of patients being admitted to and staying in hospital as inpatients. By Jan 1, 2017, 44 037 (8·6%) participants had died, 4781 (<1%) were lost to follow-up, and 27 903 (5·4%) developed cancer, including 3464 gastric cancers (International Classification of Disease 10 [ICD-10]: C16). For any reported cancer cases, systematic validation was done through a review of the original medical records (including histopathological reports) retrieved from hospitals. Among the 1355 reported gastric cancer cases that were adjudicated, 1246 (92%) were confirmed, and among the confirmed cases with pathology results available, 1060 (85%) of 1246 were adenocarcinomas.

### Design of case-cohort study

To reduce the potential effects of lower *H pylori* antibody concentrations due to gastric atrophy, the selection of incident gastric cancer cases were confined to participants who were alive and with no history of cancer 2 years after study entry. All 437 reported or confirmed CGC cases (ICD-10: C16·0) were selected, and we randomly selected 500 cases from the 762 confirmed NCGC (of 2035 reported) cases. Since CGC arises in the oesophageal-gastric junction and is often difficult to distinguish from oesophageal adenocarcinoma, which is rare in China, we also included all confirmed oesophageal adenocarcinoma cases (n=27). A subcohort of 500 participants was sampled from the modified baseline cohort (ie, participants who were alive with no history of cancer 2 years after study entry) using stratified random sampling with the sizes of the strata based on the proportions of age categories and sex of all selected participants with gastric cancer (appendix p 2). This modified baseline subcohort included approximately 100 000 previously genotyped participants who were randomly selected from the whole cohort.

### Immunoblot assay

A qualitative western blot kit assay, HelicoBlot 2.1 (MP Diagnostics; Bordeaux, France), was used to detect IgG antibodies to *H pylori* proteins in the baseline plasma samples retrieved from storage in liquid nitrogen. The assay detects seven diagnostic antibodies binding to *H pylori* proteins separated on a nitrocellulose membrane and has been shown to have a sensitivity of 96% and a specificity of 93% for the detection of *H pylori* infections in populations in Europe.^16^ The immunoblot assay results were independently classified and reviewed by two laboratory staff (one of which was DC) who were masked to the baseline information and case status of the samples. Each *H pylori* biomarker band was classified as positive, negative, or ambiguous. These results were then used to define *H pylori* seropositivity according to two different criteria. The clinical criteria, as defined by the manufacturer of the western blot kit, were the presence of one or more of the 89kD (vacuolating cytotoxin), 37kD, or 35kD bands; or both the 30kD (urease enzyme light subunit) and 19·5kD bands; or both the 116kD (cytotoxin-associated antigen) and the current infection marker bands. For this study, we modified the criteria by removing the requirement to have the current infection marker band alongside the cytotoxin-associated antigen band (ie, the cytotoxin-associated antigen band alone can define seropositivity). This choice was made for two reasons: first, the current infection marker band is more likely to be absent in patients with atrophic stomach or precancerous lesions; and second, the presence of antibodies to cytotoxin-associated antigen is the most sensitive marker of a past infection,^17^ which makes it relevant for investigating the role of *H pylori* infection in patients progressing towards cancer. These modified criteria are thereafter referred to as epidemiological criteria (appendix p 3).

### Statistical analysis

Seroprevalence estimates and their 95% CIs were calculated among the NCGC, CGC, and subcohort groups. Spearman's correlations between *H pylori* biomarkers were calculated. Cox proportional hazards models were fitted using the pseudo-partial likelihood for the Borgan III estimator^18^ to estimate hazard ratios (HRs) for risk of NCGC, CGC, and oesophageal adenocarcinoma associated with *H pylori* infection, both overall and by individual *H pylori* biomarkers, with adjustments for age (numeric), sex, individual study regions, and education (six categories: no formal school, primary school, middle school, high school, technical school or college, and university). Time in the study (time from 2 years after entry into the study to cancer diagnosis, loss to follow-up, death because of other causes, or Jan 1, 2017, whichever occurred first) was used as the timescale in the model. Models were also fitted additionally and sequentially adjusting for other gastric cancer risk factors, including smoking, alcohol drinking, a family history of cancer, body-mass index, and the consumption of some dietary factors (eg, preserved vegetables or fresh fruits). We fitted Cox regression models with piecewise constant time-varying coefficients using the Prentice estimator, with stratification by the design stratification variables (age groups and sex) and adjustment for age (years), individual study regions, and education. We additionally assessed the proportional hazards assumption using Schoenfeld residuals.

We did sensitivity analyses by assuming that all ambiguous bands were either seropositive or seronegative. Further analyses were done in subgroups defined by age (30–59 and 60–79 years), sex, area (rural or urban), education (two groups: no formal education or primary school, and middle school or higher), and body-mass index (<25 or ≥25 kg/m^2^); and among men only, by regular smoking and alcohol drinking. HRs were also calculated for different gastric cancer histopathological subtypes and by the time since blood collection (individuals with ambiguous *H pylori* status were excluded from this analysis). To facilitate comparisons with previous studies, additional sensitivity analyses were done using the clinical criteria to define *H pylori* seropositivity.

Population attributable fractions (PAFs) were calculated as PAF=P_c_ (R–1) / R, where P_c_ is the observed seroprevalence among the cases and R is the estimated HR associated with *H pylori* infection in the study.^2^ The number of new gastric cancer cases attributable to *H pylori* infection was estimated by multiplying the PAF by the national incidence rates of gastric cancer in China in 2018.^19^ Statistical analysis was done with R (version 4.0.2).

### Role of the funding source

The funder of the study had no role in study design, data collection, data analysis, data interpretation, or writing of the report.

## Results

After a median 10·1 years (IQR 9·2–11·1) of follow-up, the incidence in the whole cohort was 57·6 per 100 000 person-years for NCGC and 10·5 per 100 000 person-years for CGC, both of which increased with age. In each age group, men had a nearly 3 times higher rate than did women (appendix p 4). One case of CGC and one case of NCGC had missing *H pylori* data and were not included in analyses.

Compared with the subcohort participants, participants with NCGC were more likely to be urban residents; to have a higher socioeconomic status (measured by both education and household income); to consume meat, dairy products, and fresh fruits; to be regular smokers or alcohol drinkers (among men); and to report good self-reported health at baseline (table 1). For participants with CGC, however, the converse appeared to be true, except for preserved vegetables and spicy food, for which both cancer groups showed similar patterns to each other compared with subcohort participants (table 1). Because of the higher retrieval rates of medical notes in urban than in rural hospitals, disproportionally more participants with NCGC were selected from urban areas, which is reflected in the slightly better socioeconomic and health status among the selected participants with NCGC compared with those not selected for the study (appendix p 5).

**Table 1:** Baseline characteristics of patients with gastric cancer (NCGC and CGC) and subcohort participants

		**NCGC (n=499)**	**CGC (n=436)**	**Subcohort (n=500)**
**Demographic factors**				
Age (years)		59·0 (9·5)	61·2 (8·6)	59·1 (9·9)
Sex				
	Men	327 (66%)	325 (75%)	347 (69%)
	Women	172 (34%)	111 (25%)	153 (31%)
Urban residents		351 (70%)	159 (36%)	252 (50%)
≥6 years of education		225 (45%)	141 (32%)	217 (43%)
Household income (>¥20 000 per year)		231 (46%)	132 (30%)	210 (42%)
**Lifestyle factors**				
Current regular smoking				
	Men	230 (70%)	186 (57%)	202 (58%)
	Women	5 (3%)	0	6 (4%)
Current regular alcohol drinking				
	Men	134 (41%)	89 (27%)	111 (32%)
	Women	2 (1%)	0	7 (5%)
Physical activity (MET-h per day)		19·5 (15·2)	18·1 (15·5)	17·9 (14·0)
Medical history and health status				
	Poor self-rated health at baseline	46 (9%)	53 (12%)	55 (11%)
	Diabetes (self-reported or screen detected)	32 (6%)	25 (6%)	44 (9%)
	A history of peptic ulcer	31 (6%)	23 (5%)	27 (5%)
	A history of cirrhosis or chronic hepatitis	3 (1%)	3 (1%)	6 (1%)
	A history of CHD, stroke, or TIA	28 (6%)	31 (7%)	52 (10%)
	A history of emphysema or bronchitis	12 (2%)	18 (4%)	21 (4%)
**Anthropometry and blood pressure**				
Body mass index (kg/m^2^)		23·6 (3·4)	24·0 (3·5)	23·7 (3·5)
	Underweight (<18·5 kg/m^2^)	25 (5%)	14 (3%)	26 (5%)
	Normal (18·5–24·9 kg/m^2^)	314 (63%)	266 (61%)	301 (60%)
	Overweight (25·0–29·9 kg/m^2^)	136 (27%)	135 (31%)	150 (30%)
	Obese (≥30 kg/m^2^)	24 (5%)	21 (5%)	23 (5%)
Waist circumference (cm)		81·1 (10·1)	82·3 (10·2)	81·4 (10·3)
SBP (mm Hg)		135·3 (22·8)	138·0 (23·1)	135·8 (22·5)
**Daily dietary consumption**				
Rice		337 (68%)	183 (42%)	358 (72%)
Wheat		221 (44%)	271 (62%)	188 (38%)
Meat		174 (35%)	69 (16%)	150 (30%)
Poultry		6 (1%)	0	2 (0%)
Dairy		86 (17%)	41 (9.4)	64 (12.8)
Fresh vegetables		474 (95%)	424 (97%)	481 (96%)
Soybean		24 (5%)	14 (3%)	18 (4%)
Preserved vegetables		160 (32%)	105 (24%)	86 (17%)
Fresh fruit		125 (25%)	32 (7%)	97 (19%)
Spicy food		86 (17%)	41 (9%)	122 (24%)

Data are mean (SD) or number (%). CGC=cardia gastric cancer. CHD=coronary heart disease. MET-h=metabolic equivalent of task-hours. NCGC=non-cardia gastric cancer. SBP=systolic blood pressure. TIA=transient ischaemic attack.

Overall, the seroprevalence of *H pylori* infection was 94·4% (95% CI 92·4–96·4) in participants with NCGC, 92·2% (89·7–94·7) in participants with CGC, and 75·6% (71·8–79·4) in subcohort participants, with a further 12 (2·4%) of those with NCGC, 7 (1·6%) of those with CGC, and 21 (4·2%) in the subcohort participants classified as seroambiguous (appendix p 6). In the subcohort, the seroprevalence of *H pylori* infection was higher among urban or highly educated participants, but did not vary substantially by age or sex (appendix p 7). Among the seven biomarkers measured, cytotoxin-associated antigen had the highest seroprevalence in each study group (465 [93·2%, 95% CI 91·0–95·4] for NCGC, 400 [91·7%, 89·2–94·3] for CGC, and 370 [74·1%, 70·3–78·0] for the subcohort; appendix p 6). Moderate correlations between different *H pylori* biomarkers were observed (eg, *r*=0·58 between cytotoxin-associated antigen and vacuolating cytotoxin, and *r*=0·48 between cytotoxin-associated antigen and urease enzyme light subunit; appendix p 8).

**Figure 1:**
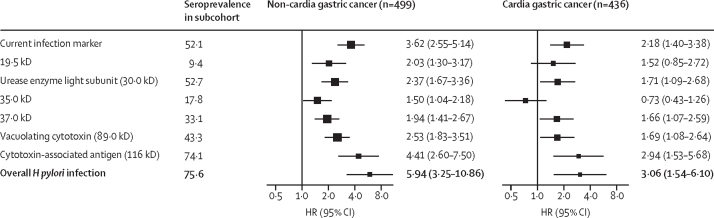
Associations of *Helicobacter pylori* infection, overall and by individual biomarkers, with risks of non-cardia gastric cancer and cardia gastric cancer *H pylori* infection was established using epidemiological criteria. Cox regression, with the time in the study as the timescale fitted using the Borgan III estimator, was used to estimate HRs, with adjustment for age, sex, area, and education. The black squares represent the adjusted HRs, with the area inversely proportional to the variance of the log HRs, and the horizontal lines representing their corresponding 95% CIs. HR=hazard ratio.

Compared with *H pylori* seronegative participants, those who were seropositive had adjusted HRs of 5·94 (95% CI 3·25–10·86) for NCGC and 3·06 (1·54–6·10) for CGC (figure 1). Additional adjustments for other risk factors did not materially alter the risk estimates (appendix p 9). The HRs did not differ significantly between women and men for both NCGC (16·96 *vs* 4·25) and CGC (7·16 *vs* 2·55) or between younger and older age groups (appendix p 10). Furthermore, the HRs did not differ significantly in population subgroups defined by other factors such as area, education, smoking, alcohol drinking, body-mass index, and family history of cancer (appendix p 10).

In sensitivity analyses using the clinical criteria to define *H pylori* infection, the seroprevalence in the NCGC group was 418 (84%) of 499, in the CGC group, 347 (80%) of 436, and in the subcohort, 288 (58%) of 500, with adjusted HRs of 4·68 (95% CI 3·13–6·98) for NCGC and 2·36 (1·42–3·94) for CGC (appendix p 11).

Among the seven individual biomarkers, cytotoxin-associated antigen showed the strongest association with the risks of NCGC and CGC, with HRs of 4·41 (95% CI 2·60–7·50) for NCGC and 2·94 (1·53–5·68) for CGC (figure 1). The other biomarkers were each associated with an approximately 2 times increased risk of both cancers, except for a null association of the 35·0 kD band with CGC risk (figure 1). For both NCGC and CGC there were also indications of positive dose–response associations with the number of positive *H pylori* biomarkers. Compared with individuals who were seronegative for all relevant biomarkers, individuals who were seropositive for both cytotoxin-associated antigen and vacuolating cytotoxin had HRs of 6·29 (95% CI 3·56–11·12) for NCGC and 3·23 (1·59–6·56) for CGC, whereas for those who were seropositive for both cytotoxin-associated antigen and urease enzyme light subunit, the HRs were 5·01 (2·70–9·29) for NCGC and 2·54 (1·20–5·39) for CGC. Being seropositive for cytotoxin-associated antigen, vacuolating cytotoxin, and urease enzyme light subunit did not further increase the risk estimates (appendix p 12).

For oesophageal adenocarcinoma, although the adjusted HRs for overall or individual biomarkers seropositivity were generally similar to those for CGC, the 95% CIs were wide because of the small number of cases involved (appendix p 13).

Although no statistically significant time interaction was detected, for both NCGC and CGC the HRs at 3–4 years were approximately half of those at 5–6 or 7 years or more since sample collection (table 2). The associations did not differ significantly between major histopathological subtypes of cancer (appendix p 14).

**Table 2:** Adjusted HRs for NCGC and CGC associated with *Helicobacter pylori* infection stratified by time since blood sample collection

	**Number of cases**	HR (95% CI)*
**NCGC**		
3–4 years	123	3·12 (1·38–7·07)
5–6 years	156	7·55 (2·66–21·45)
≥7 years	208	8·05 (3·12–20·75)
**CGC**		
3–4 years	97	1·78 (0·74–4·26)
5–6 years	109	5·11 (1·59–16·35)
≥7 years	223	3·24 (1·52–6·87)

CGC=cardia gastric cancer. HR=hazard ratio. NCGC=non-cardia gastric cancer.

* HRs estimated using Cox regression fitted using the Prentice estimator, with stratification by the design stratification variables (age groups and sex) and adjustment for age (years), area, and education.

Compared with seronegative participants, those with ambiguous *H pylori* infection status had adjusted HRs of 4·08 (95% CI 1·56–10·66) for NCGC and of 0·99 (95% CI 0·23–4·21) for CGC. When classifying all ambiguous results as seropositive or seronegative, the HRs were attenuated for both NCGC and CGC (appendix p 15).

In this population, we estimated that the PAF for *H pylori* was 78·5% for NCGC and 62·1% for CGC. We estimated that based on the 2018 China cancer statistics,^19^ *H pylori* infection would have caused approximately 339 955 new cases of gastric cancer (271 389 NCGC cases and 68 566 CGC cases) in China annually (table 3).

**Table 3:** The estimated numbers of cases attributable to *Helicobacter pylori* in China

	**Seropositive (%, 95% CI)**	HR (95% CI)*	**Population attributable fractions (%)**	**Attributable cases**
NCGC	471/499 (94·4%, 92·4–96·4%)	5·94 (3·25–10·86)	78·5%	271 389
CGC	402/436 (92·2%, 89·7–94·7%)	3·06 (1·54–6·10)	62·1%	68 566

CGC=cardia gastric cancer. HR=hazard ratio. NCGC=non-cardia gastric cancer.

* HRs estimated using the Borgan III estimator and adjusted for age (years), sex, individual study regions, and education.

## Discussion

This large prospective case-cohort study shows clearly that *H pylori* infection was a strong risk factor not only for NCGC but also for CGC in Chinese adults. Using a sensitive immunoblot assay together with the exclusion of the first 2 years after sample collection, we showed that *H pylori* infection was associated with a 6 times higher risk of NCGC and a 3 times higher risk of CGC. It is likely that the observed associations are largely causal, hence *H pylori* infection would have caused approximately 80% of NCGC and more than 60% of CGC in this population.

**Figure 2:**
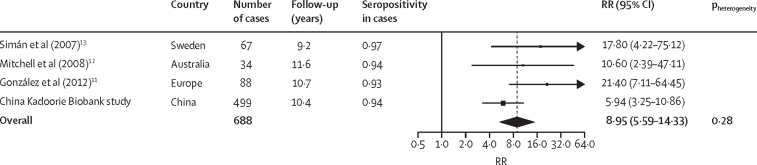
Meta-analysis of the association of *Helicobacter pylori* with non-cardia gastric cancer in the China Kadoorie Biobank study and three published studies using the same immunoblot assay The combined estimate is an inverse-variance weighted average. The black squares represent RR, with the area inversely proportional to the variance of the log RR, and the horizontal lines represent their corresponding 95% CIs. The dotted vertical line indicates the overall RR, and the black diamond indicates it and its 95% CI. p_heterogeneity_ denotes the p value for heterogeneity across the four studies. RR=relative risk.

Previous studies of *H pylori* infection and gastric cancer were mainly based on the less sensitive ELISA^5^, ^7^, ^9^, ^20^ or, more recently, multiplex serology assays.^21^, ^22^, ^23^, ^24^ Although an increased risk of NCGC with *H pylori* has been shown consistently, the magnitude of the association varied greatly between studies, and was generally smaller in Chinese populations than in populations in Europe, the USA, and Australia. In a pooled analysis of 12 prospective studies,^5^ *H pylori* infection established by ELISA was associated with an odds ratio (OR) of 3·0 (95% CI 2·3–3·8) for NCGC (n=762 cases), increasing to 5·9 (3·4–10·3) among individuals with blood samples collected more than 10 years before cancer diagnosis (n=223 cases). There is evidence from studies in Europe and Australia that the use of immunoblot assay leads to greater risk estimates than those based on ELISA. In the Eurogast-EPIC study involving 88 NCGC cases and 338 controls,^11^ the OR for NCGC was 21·4 (95% CI 7·1–64·4) using similar seropositivity criteria to those in the present study, which was three times as high as that based on ELISA (6·8 [95% CI 3·0–15·1]). Similar but less striking differences were also shown in another two small studies comparing immunoblot assays with ELISA assays in the same study populations.^12^, ^13^ Overall, these three studies in Europe and Australia based on immunoblot assays with a total of just 189 participants with NCGC yielded a pooled RR of 17·0 (95% CI 11·6–25·0),^6^ which has been used to estimate the global burden of NCGC attributable to *H pylori* infection.^2^ For comparison, in our study using the same immunoblot assay and with more than 2·5 times as many participants as in all the three studies combined, the RR estimate for NCGC was only approximately a third of those of the previous studies. The reasons for such a large difference in the risk estimates are not fully understood, but might reflect the differences in the background prevalence of *H pylori* infection in China (75% in the China Kadoorie Biobank study *vs* 59% in Eurogast-EPIC^11^), and potentially a comparatively more notable role of other factors in the cause of gastric cancer in Chinese populations than other populations. Although not significant, we observed higher HRs in women, who rarely smoked or drank alcohol, than in men. In a combined analysis of the present study and the published data from these three studies in Europe and Australia,^6^ the pooled RR was 8·95 (5·59–14·33; figure 2), and there was no significant heterogeneity in the risk estimates from these four different studies. This updated risk estimate for NCGC should help inform the future estimation of the disease burden associated with *H pylori* infection, both regionally and globally.

The existing evidence linking CGC with *H pylori* infection remains conflicting, with studies from Europe, the USA, and Australia generally reporting null or inverse associations, whereas studies from east Asian countries tended to show modest positive associations. CGC is defined as a lesion with its centre located within 1 cm proximal and 2 cm distal to the oesophageal-gastric junction. However, there might be differences in the anatomical locations of CGC lesions between different populations, with CGC in populations in Europe, the USA, and Australia mainly involving the distal oesophagus, and in east Asians, mainly involving the proximal stomach.^7^, ^9^ In the aforementioned pooled analyses of 12 prospective studies (eight in Europe, the USA, and Australia and four in east Asian countries), overall there was no clear association of *H pylori* infection with CGC (274 cases; OR 0·99 [95% CI 0·72–1·35]), with one study in China (n=99) showing a significant 77% excess risk.^5^ In another meta-analysis of 30 studies (ten prospective and 20 retrospective),^9^ there was a significantly lower risk of CGC (OR 0·78, 95% CI 0·63–0·97) in 16 studies in Europe, the USA, and Australia but a significantly higher risk of CGC with *H pylori* infection (RR 1·98, 95% CI 1·38–2·83) in 14 east Asian studies, driven mainly by large RRs in retrospective studies. The present study provides important new evidence about the role of *H pylori* infection in the cause of CGC in the Chinese population. Our RR estimates are approximately twice those in previous studies of Chinese and other Asian populations that used less sensitive assays and suboptimal study designs.^4^, ^7^, ^21^, ^22^, ^23^, ^24^ Our findings are also consistent with the notion that in east Asians, CGC might mainly involve the proximal stomach.^7^, ^9^ Moreover, the associations were similar when the analyses were further confined to clinically adjudicated CGC cases (appendix p 16). Furthermore, the association of *H pylori* with oesophageal adenocarcinoma, which is rare in the Chinese population, was similar to the association with CGC. Further studies are warranted to further substantiate (or refute) the seemingly discrepant role of *H pylori* in the cause of CGC between populations in east Asia and in Europe, the USA, and Australia.

There is evidence that the development of atrophic gastritis occurs in late stage pre-malignant cases and not accounting for this occurrence might result in an underestimation of the risk associated with *H pylori* infection.^25^, ^26^ Indeed, our study showed that although there was no evidence that the proportional hazards assumption is inconsistent with the data, the RRs of both NCGC and CGC during the early period of follow-up (even after excluding the first 2 years of follow-up as part of our study design) tended to be lower than those during the subsequent years of follow-up. This result suggests that the exclusion of the first 2 years of follow-up might have not fully accounted for this occurrence. If this assumption were true, the real risk estimates (and the attributable fractions) for both NCGC and CGC associated with *H pylori* infection would be higher (appendix p 17).

A few studies have also shown the predominant role of cytotoxin-associated antigen-positive *H pylori* strains, in which nearly all participants with NCGC were cytotoxin-associated antigen-positive.^11^, ^12^ Among all *H pylori* biomarkers, antibodies to cytotoxin-associated antigen have the strongest immunoreactivity and are the last to disappear after *H pylori* eradication or in the precancerous stomach.^25^ As such, the epidemiological criteria used in the present study (and the Eurogast-EPIC study^11^) should provide a good indicator of past infection. Consistent with previous studies of populations in Europe, the USA, and Australia,^4^, ^13^, ^23^, ^27^, ^28^ our study showed that cytotoxin-associated antigen had the strongest associations with the risks of both NCGC and CGC of all the measured biomarkers among Chinese adults. Moreover, there were also higher RRs associated with larger numbers of other specific biomarkers (eg, vacuolating cytotoxin and urease enzyme light subunit) in addition to cytotoxin-associated antigen.

Apart from the prospective study design and inclusion of the large number of well characterised cases, the key strengths of our study were the use of a sensitive immunoblot assay, the exclusion of the first few years of follow-up to reduce the possible effects of atrophic gastritis on seropositivity, the ability to adjust for many known or potential risk factors for gastric cancer, and for individual study regions to account for large regional variations in *H pylori* seroprevalence and cancer rates.^16^ Furthermore, our study was the first to show separate results from seroambiguous bands; misclassification of those as seropositive or seronegative is likely to bias the risk estimates. However, the study also has limitations. First, *H pylori* has approximately 1500 proteins,^10^ and the HelicoBlot 2.1 assay only covers seven of them. Hence, the effects of other *H pylori* proteins and their interactions could not be investigated. Moreover, the immunoblot assay used was developed on the basis of the *H pylori* strains typically seen in populations in Europe, the USA, and Australia, with no available data for separate validation nor direct comparison with conventional ELISA assays in Chinese populations. Second, because of the high retrieval rates of medical notes in urban hospitals, more participants with NCGC in urban areas were selected in our study. However, with comprehensive adjustment for confounding, this difference is unlikely to affect the RR estimates. For participants with CGC, given the low proportion of adjudicated cases, we also included reported cases, all of which were specifically ICD-10 coded as C16·0 when first reported, thus minimising the risk of misclassification. Third, we did not investigate simultaneously the role of other pathogens (eg, Epstein-Barr virus) and their potential interactions with *H pylori* in the causation of gastric cancer. Lastly, although various measures were taken in analyses, residual confounding and other unknown biases might persist.

In summary, the present study provides robust new evidence about the causal role of *H pylori* infection in both NCGC and CGC in China, where *H pylori* infection is widespread. The observed associations are probably largely causal and suggest that *H pylori* infection causes approximately 0·34 million gastric cancer cases annually in China. In addition to improving the estimation of the global burden of cancer because of *H pylori* infection, our study findings should also inform prevention strategies in China and elsewhere. To date, there is no prophylactic or therapeutic vaccination against *H pylori.* Moreover, despite the availability of highly cost-effective anti-*H pylori* treatments, population-based screening and eradication have not yet been included in the national cancer primary prevention programme in China. Our findings, together with evidence from a randomised controlled trial of reduced gastric cancer risk after *H pylori* eradication,^29^ suggest that population-based *H pylori* mass screening and eradication should be considered as a key strategy for gastric cancer prevention, before considering mass gastric cancer screening by barium photofluorography or endoscopy in China and high-risk settings globally.

## Data sharing

Anonymised baseline, resurvey, and cause-specific mortality and morbidity data are available for access through a formal application on the China Kadoorie Biobank website. The application will then be reviewed by a Data Access Committee. Further details about the access policy and procedures can be found on the website.

For the **China Kadoorie Biobank website** see www.ckbiobank.org

## Declaration of interests

We declare no competing interests.
