# Supplementary material for: The relative and attributable risks of cardia and non-cardia gastric cancer associated with Helicobacter pylori infection in China: a case-cohort study
Source: Lancet Public Health. 2021 Nov 25;6(12):e888–96. doi: 10.1016/S2468-2667(21)00164-X (PMC8646857; doi:10.1016/S2468-2667(21)00164-X)
Supplement: Supplementary appendix [file mmc1.pdf]

THE LANCET
Public Health

Supplementary appendix

This appendix formed part of the original submission and has been peer reviewed. We post it as supplied by the authors.

Supplement to: Yang L, Kartsonaki C, Yao P, et al. The relative and attributable risks of cardia and non-cardia gastric cancer associated with *Helicobacter pylori* infection in China: a case-cohort study. *Lancet Public Health* 2021; **6:** e888–96.

SUPPLEMENTARY APPENDIX

The relative and attributable risks of non-cardia and cardia gastric cancer associated with *H. pylori* infection in China: A case-cohort study. Yang L, Kartsonaki C, Pang Y, et al.

Content List of Supplementary Materials **Page**

**eFigure1.** Flow diagram of study design and participant selection	2
**eTable 1.** Criteria for determining *H. pylori* sero-positivity based on the HelicoBlot assay	3
**eTable 2.** Incidence rates of NCGC and CGC by sex and age in the whole CKB cohort	4
**eTable 3.** The baseline characteristics of NCGC cases selected and not selected for the present study	5
**eTable 4.** Sero-status of *H. pylori* biomarkers and *H. pylori* infection in each study arm	6
**eTable 5.** *H. pylori* sero-status in each study arm by age, sex, area and education	7
**eFigure 2.** Correlation coefficients of different *H. pylori* biomarkers among the 500 subcohort participants	8
**eFigure 3.** Associations of *H. pylori* infection with risks of (a) non-cardia, and (b) cardia gastric cancer, with further stepwise adjustments for other risk factors	9
**eFigure 4.** Associations of *H. pylori* infection with risks of (a) non-cardia, and (b) cardia gastric cancer, in selected population subgroups	10
**eTable 6.** Sero-prevalence and associations of *H. pylori* infection, defined by ‘clinical criteria’, with risks of gastric cancer	11
**eFigure 5:** Associations of sero-positivity of multiple key *H. pylori* biomarkers with risks of (a) non-cardia, and (b) cardia gastric cancer	12
**eFigure 6.** Associations of *H. pylori* infection, overall and by individual biomarkers, with risk of oesophageal adenocarcinoma	13
**eTable 7.** Associations of *H. pylori* infection with risks of non-cardia and cardia gastric cancer by histopathological subtypes	14
**eFigure 7.** Associations of *H. pylori* infection with risks of non-cardia and cardia gastric cancer and oesophageal adenocarcinoma, with sero-ambiguous results included either as a) sero-negative, or b) sero-positive	15
**eFigure 8.** Associations of *H. pylori* infection, overall and by individual biomarkers, with risk of cardia gastric cancer (adjudicated cases only)	16
**eTable 8.** The estimated numbers of cases attributable to *H. pylori* in China, from incremental exclusion of follow-up years data	17

eFigure1. Flow diagram of study design and participant selection

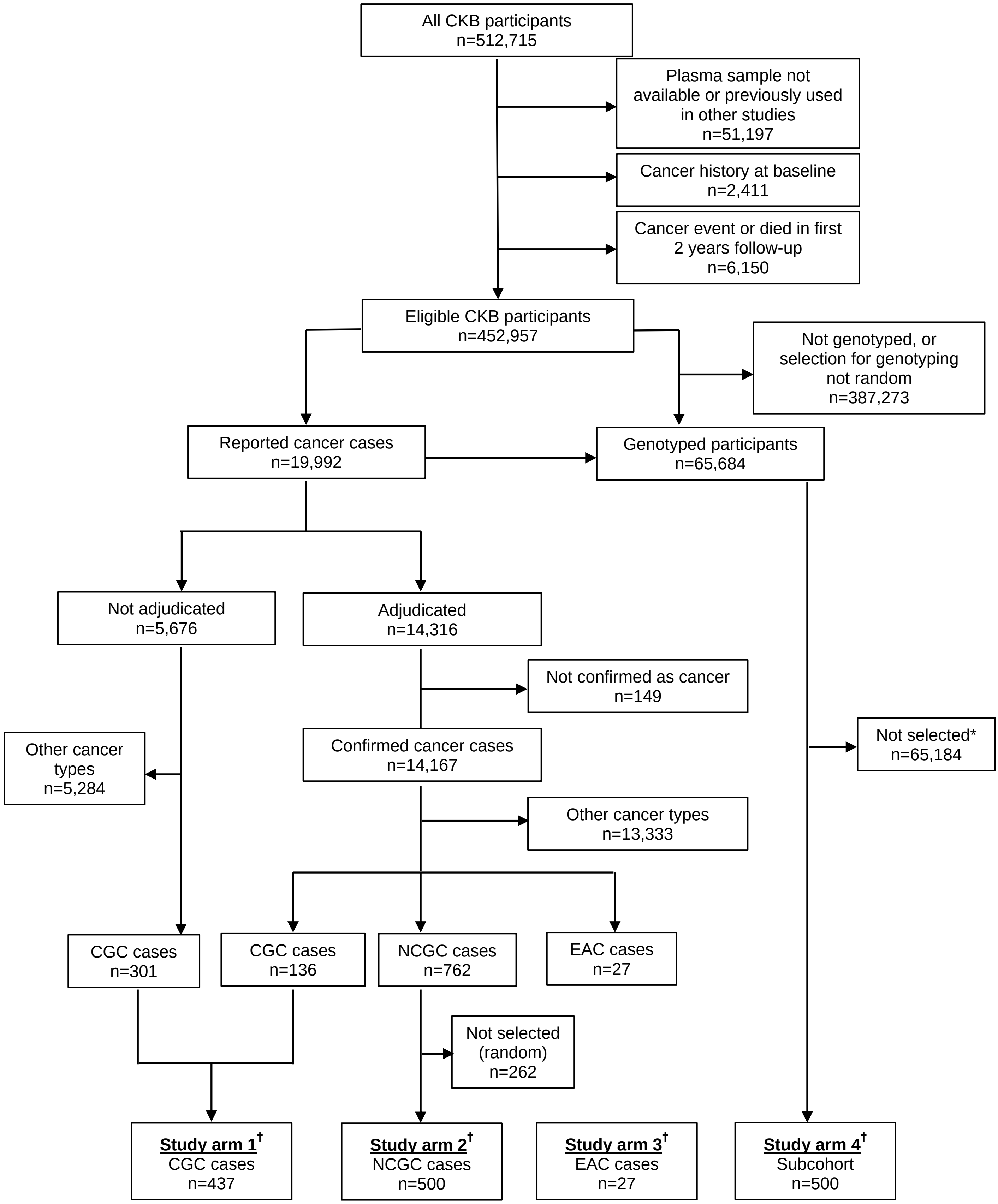

*Selection for the subcohort used stratified random sampling based on the age and sex distribution of all selected CGC and NCGC cases combined; †Individuals may be included in more than one study arm; CKB: China Kadoorie Biobank; CGC: cardia gastric cancer; NCGC: non-cardia gastric cancer; EAC: oesophageal adenocarcinoma

eTable 1. Criteria for determining *H. pylori* sero-positivity* based on the HelicoBlot assay

Epidemiological criteria		Clinical criteria
VacA, 37kD or 35kD		VacA, 37kD or 35kD
	**OR**	
UreA and 19.5kD		UreA and 19.5kD
	**OR**	
CagA		CagA and Current Infection Marker (CIM)

* If one or more bands are assessed as ambiguous (i.e. cannot be clearly determined as present or absent), this may result in an ambiguous assignment of *H. pylori* sero-status

eTable 2. Incidence rates (per 100,000 person-years) of NCGC and CGC by sex and age in the whole CKB cohort

Sex	Age (Years)	NCGC		CGC	
		No. of cases	Rate	No. of cases	Rate
**Male**	30-49	249	27.6	37	4.1
	50-59	622	99.2	132	21.1
	60-79	1032	200.4	233	45.3
**Female**	30-49	206	14.2	11	0.8
	50-59	324	34.2	49	5.2
	60-79	496	78.0	73	11.5
**Overall**		**2929**	**57.6**	**535**	**10.5**

eTable 3. The baseline characteristics of NCGC cases selected and not selected for the present study

Characteristics*	Selected	Not selected
Number of cases	500	1535
Age (years), mean (SD)	59.0 (9.5)	59.5 (9.8)
Women, n (%)	173 (34.6)	575 (37.5)
Urban residents, n (%)	352 (70.4)	774 (50.4)
≥ 6 years of education, n (%)	225 (45.0)	542 (35.3)
Household income (≥ 20000 CNY/year), n (%)	232 (46.4)	525 (34.2)
Current regular smoking, n (%)		
Males	230 (70.3)	599 (62.4)
Females	5 (2.9)	22 (3.8)
Current regular alcohol, n (%)		
Males	134 (41.0)	344 (35.8)
Females	2 (1.2)	15 (2.6)
MET h/day, mean (SD)	19.5 (15.2)	18.8 (13.4)
Poor self-rated health, n (%)	46 (9.2)	196 (12.8)
Diabetes (self-reported or screen-detected), n (%)	32 (6.4)	110 (7.2)
Prior peptic ulcer, n (%)	31 (6.2)	99 (6.4)
Prior cirrhosis/chronic hepatitis, n (%)	3 (0.6)	15 (1.0)
Prior CHD/stroke/TIA, n (%)	28 (5.6)	83 (5.4)
Prior emphysema/bronchitis, n (%)	12 (2.4)	52 (3.4)
BMI (kg/m^2), mean (SD)	23.6 (3.4)	23.2 (3.4)
Waist circumference (cm), mean (SD)	81.1 (10.1)	80.1 (10.0)
SBP (mmHg) , mean (SD)	135.3 (22.8)	135.4 (22.3)

eTable 4. Sero-status of *H. pylori* biomarkers and *H. pylori* infection (%) in each study arm

***H. pylori* infection**	**NCGC (n=499)**			**CGC (n=436)**			**Subcohort (n=500)**		
	**Positive**	**Negative**	**Amb.***	**Positive**	**Negative**	**Amb.***	**Positive**	**Negative**	**Amb.***
CIM	78.6	16.8	4.6	74.5	22.5	3.0	52.1	42.9	5.0
19.5kD	16.2	78.4	5.4	14.4	81.2	4.4	9.4	87.4	3.2
30kD (UreaseA)	71.3	20.0	8.6	64.9	24.3	10.8	52.7	39.3	8.0
35kD	22.0	69.3	8.6	17.9	73.2	8.9	17.8	77.2	5.0
37kD	50.1	40.1	9.8	45.0	44.0	11.0	33.1	58.9	8.0
89kD (VacA)	64.5	25.1	10.4	59.4	29.4	11.2	43.3	47.1	9.6
116kD (CagA)	93.2	4.6	2.2	91.7	6.9	1.4	74.1	21.8	4.0
**Overall**	**94.4**	**3.2**	**2.4**	**92.2**	**6.2**	**1.6**	**75.6**	**20.2**	**4.2**

* Ambiguous results where the presence or absence of the band was not clear

eTable 5. *H. pylori* sero-status (%) in each study arm, by age, sex, area and education

	NCGC (n=499)			CGC (n=436)			Subcohort (n=500)		
	**Positive**	**Negative**	**Amb.***	**Positive**	**Negative**	**Amb.***	**Positive**	**Negative**	**Amb.***
**Age**									
30-49	96.6	2.3	1.1	95.0	5.0	0	77.1	21.4	1.4
49-59	96.7	1.7	1.7	93.2	4.7	2.0	74.1	22.4	3.4
60-79	91.8	4.8	3.5	91.1	7.3	1.6	76.2	18.4	5.5
**Sex**									
Men	93.6	4.0	2.4	92.0	6.5	1.5	76.1	19.6	4.3
Women	95.9	1.7	2.3	92.8	5.4	1.8	74.5	21.6	3.9
**Area**									
Rural	92.6	5.4	2.0	91.3	6.1	2.5	66.5	28.6	4.8
Urban	95.2	2.3	2.6	93.7	6.3	0	84.5	11.9	3.6
**Education**									
Illiterate	95.1	2.5	2.5	93.6	4.6	1.8	76.6	19.1	4.3
Primary school	90.1	6.6	3.3	93.0	5.9	1.1	65.6	26.5	7.9
Secondary school	96.9	1.6	1.6	88.9	10.1	1.0	82.8	15.6	1.6
High school or more	96.9	1.0	2.1	92.9	2.4	4.8	85.3	14.7	0
**Overall**	94.4	3.2	2.4	92.2	6.2	1.6	75.6	20.2	4.2

* Ambiguous results where the presence or absence of the band was not clear

eFigure 2. Correlation coefficients of different *H. pylori* biomarkers among the 500 subcohort participants

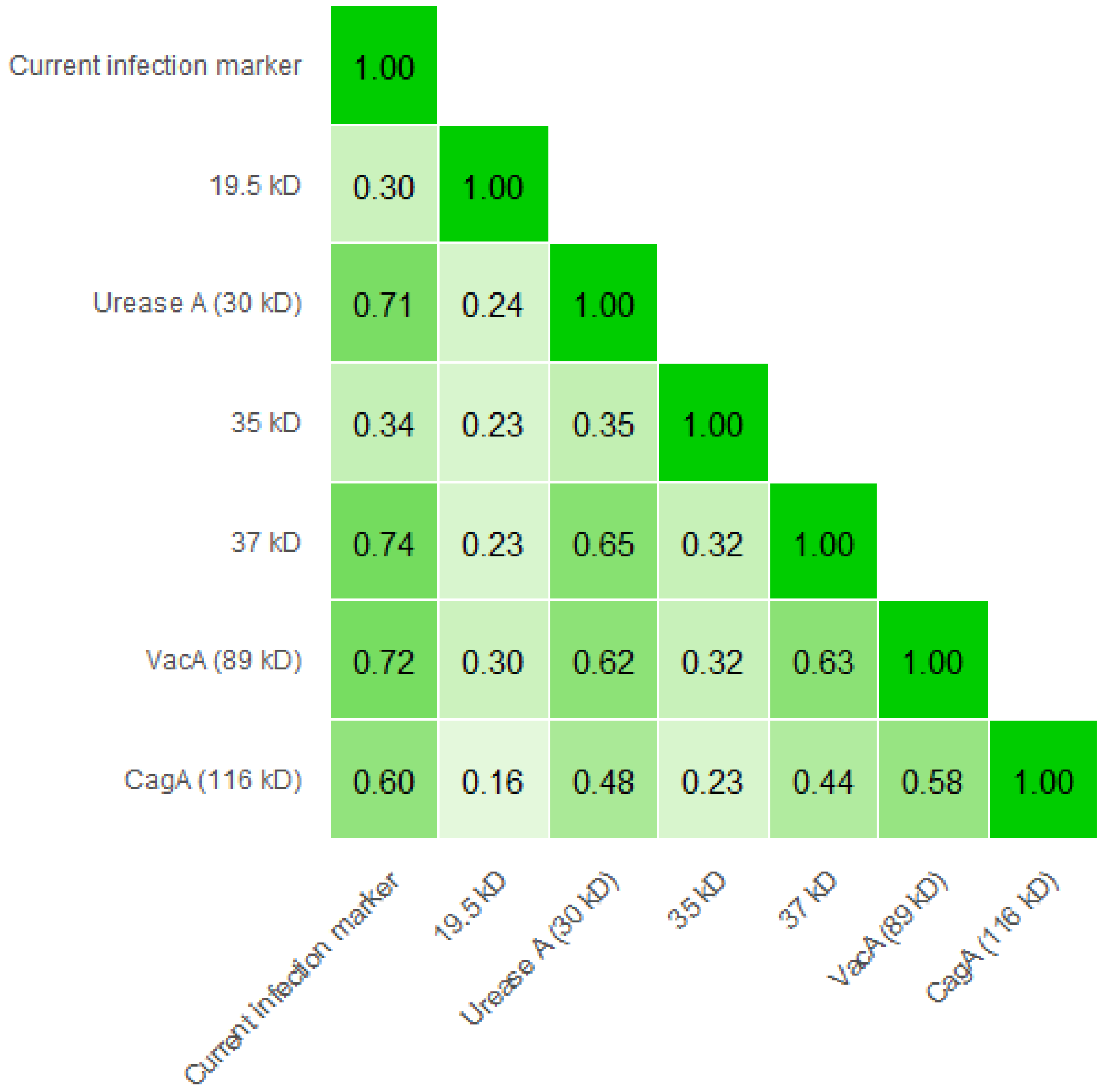

eFigure 3. Associations of *H. pylori* infection with risks of (a) non-cardia, and (b) cardia gastric cancer, with further stepwise adjustments for other risk factors

The Borgan III method was used to estimate HRs with time in study as the time scale. The squares represent adjusted HRs, and the horizontal lines represent 95% CIs. The area of the squares is inversely proportional to the variance of the log HRs. The dietary factors included preserved vegetables, fresh fruits, spicy food and soybeans.

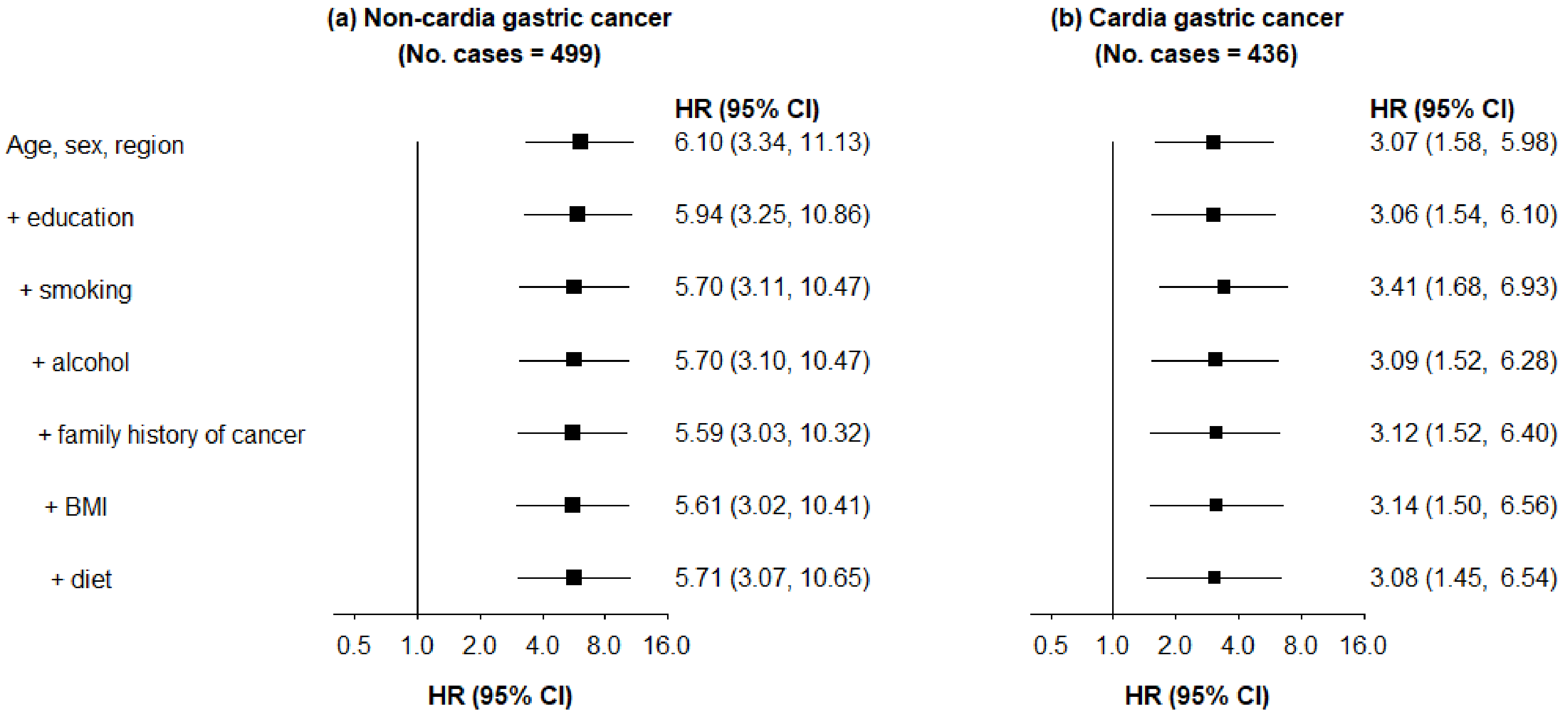

eFigure 4. Associations of *H. pylori* infection with risks of (a) non-cardia, and (b) cardia gastric cancer, in selected population subgroups

The Borgan III method was used to estimate HRs, with time in study as the time scale, and adjustment for age, sex, area and education in the model. The solid squares represent HRs, and the horizontal lines represent 95% CIs. The area of the squares is inversely proportional to the variance of the log HRs. The solid diamond represents overall HR.

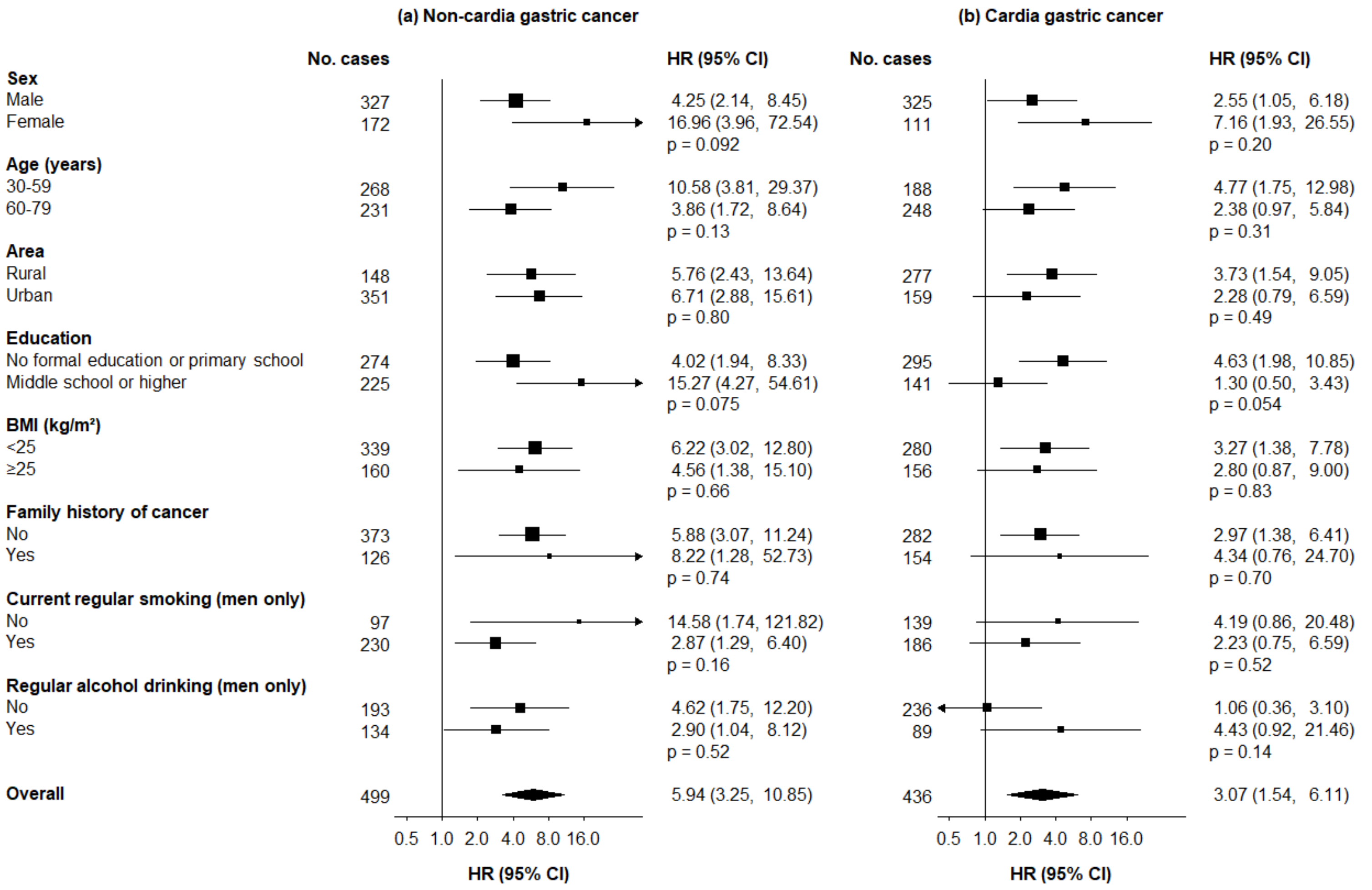

eTable 6. Sero-prevalence and associations of *H. pylori* infection, defined by 'clinical criteria', with risks of gastric cancer

Type	Sero-positive (%)	HR (95% CI) *
Subcohort	57.6	Ref.
NCGC	83.8	4.68 (3.13-6.98)
CGC	79.6	2.36 (1.42-3.94)

***** Adjusted for age, sex, area and education

eFigure 5: Associations of sero-positivity of multiple key *H. pylori* biomarkers with risks of (a) non-cardia, and (b) cardia gastric cancer

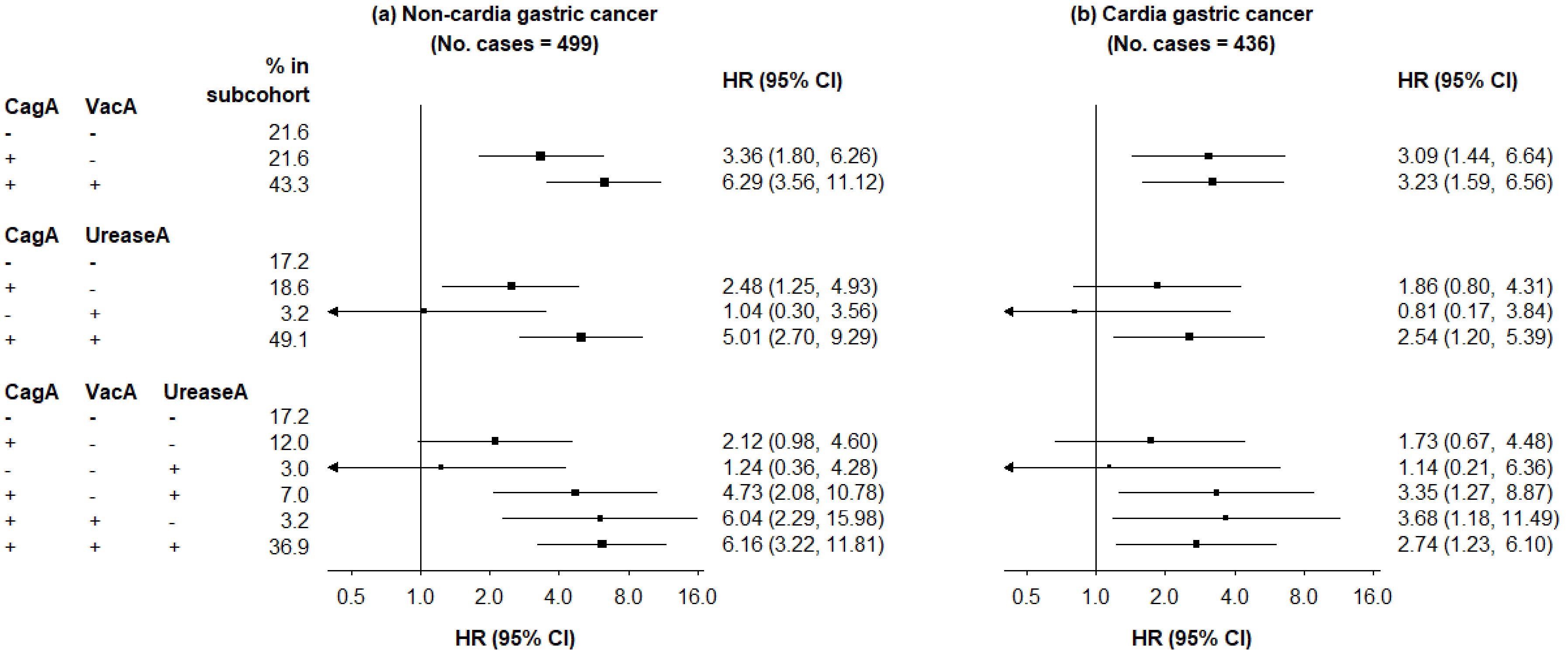

eFigure 6. Associations of *H. pylori* infection, overall and by individual biomarkers, with risk of oesophageal adenocarcinoma

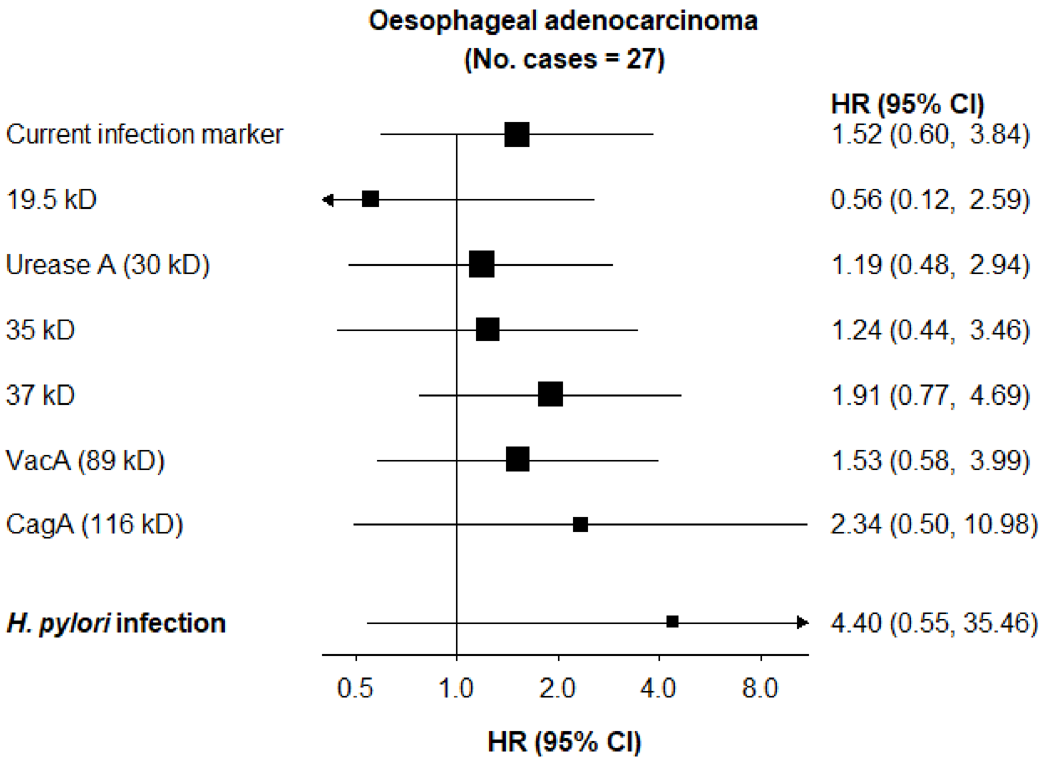

eTable 7. Associations of *H. pylori* infection with risks of non-cardia and cardia gastric cancer by histopathological subtypes

Pathological subtype	No. of cases	HR (95%CI) *
**NCGC**		
Adenocarcinoma	338	6.15 (2.95, 12.78)
Non-adenocarcinoma	48	10.03 (1.26, 80.18)
Unknown	113	4.78 (1.75, 13.02)
**CGC**		
Adenocarcinoma	98	1.91 (0.75, 4.90)
Non-adenocarcinoma	16	2.73 (0.33, 22.62)
Unknown	321	3.77 (1.72, 8.28)

***** Adjusted for age, sex, area and education

eFigure 7. Associations of *H. pylori* infection with risks of non-cardia and cardia gastric cancer and oesophageal adenocarcinoma, with sero-ambiguous results included either as (a) sero-negative, or (b) sero-positive

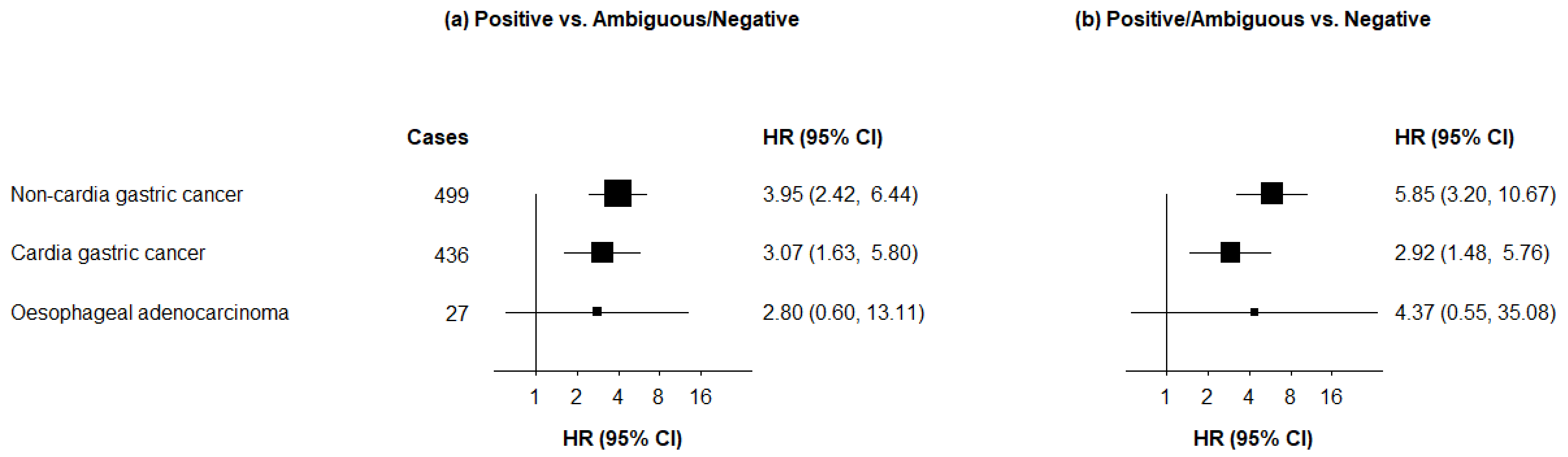

eFigure 8. Associations of *H. pylori* infection, overall and by individual biomarkers, with risk of cardia gastric cancer (adjudicated cases only)

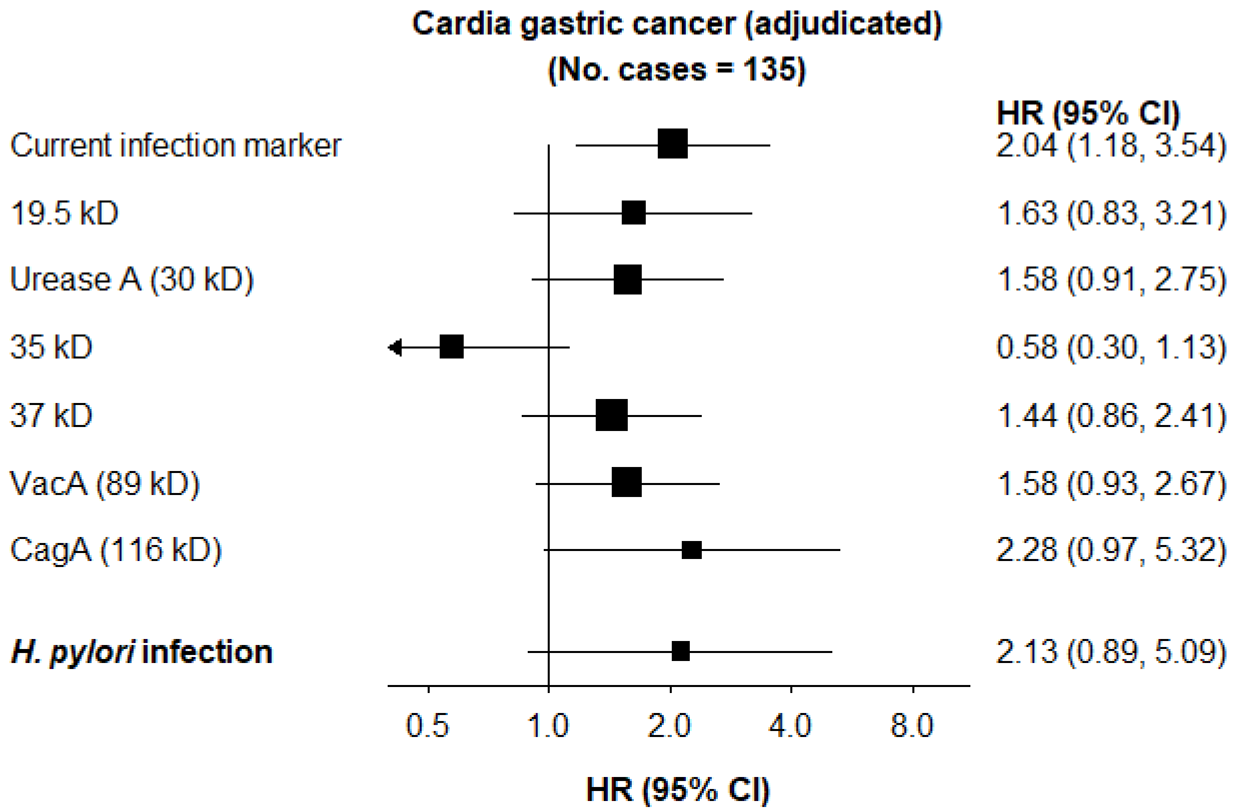

eTable 8. The estimated numbers of cases attributable to *H. pylori* in China, from incremental exclusion of follow-up years data

Follow-up years exclusion	Cancer	Sero-positive (%)	HR (95% CI)*	PAF (%)	Attributable cases
**3 years**	**NCGC**	95.6	8.62 (4.20, 17.68)	84.5	291,956
	**CGC**	93.3	3.62 (1.73, 7.55)	66.7	73,666
**4 years**	**NCGC**	95.4	8.00 (3.78, 16.95)	83.4	288,453
	**CGC**	93.4	3.68 (1.73, 7.83)	68.0	75,139
**5 years**	**NCGC**	94.7	6.12 (2.87, 13.07)	79.3	274,003
	**CGC**	92.8	3.50 (1.59, 7.69)	66.4	73,299

* HRs estimated using the Borgan III estimator and adjusted for age, sex, area and education
